# Effects of *Launaea procumbens* on brain antioxidant enzymes and cognitive performance of rat

**DOI:** 10.1186/1472-6882-12-219

**Published:** 2012-11-14

**Authors:** Rahmat Ali Khan

**Affiliations:** 1 Department of Biotechnology, Faculty of Biological Sciences, University of Science and Technology, Bannu, Pakistan

**Keywords:** Launaea procumbens, GSH, AChE, Antioxidants enzymes

## Abstract

**Background:**

*Launaea procumbens* is used in the treatment of oxidative stress and mental disorders. The effects of *Launaea procumbens* methanolic extracts (LPMEs), i.e., 100 and 200 LPME mg/kg body weight (b.w.), on cognitive performance as well as on the activity of acetylcholinesterase, and antioxidant enzymes in rat brain tissue homogenates were evaluated.

**Methods:**

Thirty male Sprague–Dawley rats were divided equally into three groups. Rats in group I (control) were given saline (vehicle), group II received LPME (100 mg/kg b.w., p.o.), and group III were treated with LPME (200 mg/kg b.w., p.o.) in dimethyl sulfoxide (DMSO) for 7 days. Antioxidant potential was assessed by measuring the activity of the antioxidant enzymes superoxide dismutase (SOD), catalase (CAT), glutathione peroxidase (GSHpx), glutathione reductase (GSR) and glutathione-S-transferase (GST) as well as lipid peroxidation and glutathione (GSH) contents in brain tissue homogenates. Activity of acetylcholinesterase (AChE) and cognitive performance were also assessed.

**Results:**

LPME administration reduced the levels of lipid peroxidation products (TBARS contents), increased GSH levels and enhanced the activities of SOD, CAT, GSHpx, GSR and GST. AChE activity was reduced by LPME treatment compared with untreated controls.

**Conclusion:**

These findings suggested the significant impact of LPMEs on brain function. These effects could be through the antioxidant effects of the bioactive constituents present in LPME.

## Background

Herbs possess bioactive constituents such as polyphenolic compounds which regulate the defense system of the plant against oxidative insult. The most important bioactive constituents of plants are alkaloids, tannins, flavonoids, and phenolic compounds [1]. Plant polyphenols possess antioxidant, antimicrobial, anticancer, allelopathic and anti-inflammatory properties. *Launaea procumbens* (LP) is a plant used in the treatment of rheumatism, inflammation and oxidative dysfunction in the kidney [2], reproductive disorders [3], hormonal imbalances [4] and liver dysfunction [5]. Nutritional analyses have shown that *Launaea procumbens* is composed of synergic acid, 2-methyl-resercinol, salicylic acid, vanillic acid, and gallic acid [6]**,** which have antioxidant, anticancer, neuroprotective and cardioprotective effects [7-9]. Previous investigations have revealed that nutrient supplementation can significantly control cognitive and motor neuron dysfunction in old age [10]. Similarly, medicinal plants and their bioactive constituents can improve behavioral (motor and cognitive behavior), neuronal signaling and anti-inflammatory effects [11]. Supplementation with *Launaea procumbens* has been shown to inhibit the production of free radicals as well as to reduce lipid peroxidation in rats [2]. Cognitive functions are regulated by the central cholinergic system and the activity of the enzyme acetylcholinesterase. Alteration of acetylcholinesterase activity is the main indicator of Alzheimer’s disease [12-14].

Therefore, the present study was designed to investigate the effect of administration of *Launaea procumbens* in rats on cognitive performance as well as the activity of acetylcholinesterase, and antioxidant enzymes in rat brain tissue homogenates.

## Methods

### Collection of LP

Whole plant of *Launaea procumbens* at maturity was collected from District Bannu during Dec 2010, after identification the plant by Prof. Dr. Mir Azab Khan, Dean Faculty of Biological Sciences and submitted their voucher specimen at the Herbarium of Biotechnology, UST Bannu, KPK, Pakistan for future reference. After shade drying and chopping, plant was ground mechanically.

### Plants extract preparation

1 kg dry powder of *Launaea procumbens* was socked in 4 liter aqueous methanol (80% methanol: 20% water) for 7 days. After one week of socking extract was filtrated using whatman filter 45. Filtrate was dried using rotary evaporator at 40 °C temperature and low pressure. The crude methanolic extract was stored at 4 ºC for *in vivo* investigations.

### *In vitro* acetylcholinesterase inhibition assay

Method of Ellman *et al.*[15] was used for assessment of AChE activity. Briefly, reaction mixture composed of 25 μl of ATCI (15mM), 75 μl of DTNB (3mM) and 50 μl of Tris- HCl, pH 8.0, (50mM), BSA (0.1%), and 25 μl of LPME was mixed and took OD at 405 nm after incubation for 5 min at room temperature. Inhibition of AChE was measured using blank in percentage. Experiments were repeated in triplicate.

### Ethical approval of the study protocol

The study protocol was approved by an Ethics Committee of Quaid-i-Azam University for the proposed study as well as Feeding and Care of Laboratory Animals.

### Animals

30 rats (180–190 g, b.w.), were provided by NIH Islamabad, Pakistan. The entire rats were placed at 25±3ºC with a half day light and dark cycle. Food and water was supplied timingly. Rats were divided randomly into three groups as;

Group 1 (Control)

Group II 100 mg /kg b.w. LPME

Group III 200 mg /kg b.w. LPME

Experiment was conducted for 7 days. At the end of the experiment all animals were sacrificed; blood was drawn prior to the excision of brain, then treated with liquid nitrogen and stored at -80°C for further enzymatic analysis.

### Behaviors study

Behaviors study (step-through passive avoidance task) was carried out using modified protocol as used by Kameyama et al. [16]. During this procedure, briefly after first training and acquisition test at 5th day rats were allowed into two chamber (light/dark) equipment with passive avoidance. At 5th day rats were allowed for the attainment test in the light compartment. After hundred seconds, animals were allowed to enter the dark chamber by opening door and recorded the latency with removing rats that reach after 100 seconds using electric shock. After 30 min the trial was repeated. Initial latency (IL) was recorded for entrance into the dark chamber. After 24 hours, rats were tested for step-through latency (measuring time into dark section) for 5 min. Experiment was repeated during 09:00am and 15:00pm.

### Estimation of oxidative status

Homogenization of brain tissue was carried out in phosphate buffer (pH 7.6), centrifuged at 20,000rpm*×g* at 4°C for 2 hour, to obtain a soluble salt part (SS). Re-extraction of the pellets was carried out to get a soluble detergent part (DS) [17]. Supernatant of both parts were stored at −20°C. BSA was used for estimation of protein with different concentrations. Activities of various antioxidant enzymes are in brain tissue homogenate were measured as.

### Catalase activity (CAT)

CAT activities were determined by the method of Chance and Maehly [18] with some modification. The reaction solution of CAT activities contained 2.5 ml of 50 mM phosphate buffer (pH 5.0), 0.4 ml of 5.9 mM H_2_O_2_ and 0.1 ml enzyme extract. Changes in absorbance of the reaction solution at 240 nm were determined after one minute. One unit of CAT activity was defined as an absorbance change of 0.01as units/min.

### Super oxide dismutase assay (SOD)

SOD activity was estimated by the method of Kakkar et al. [19]. Reaction mixture of this method contained 0.1 ml of phenazine methosulphate (186 μM), 1.2 ml of sodium pyrophosphate buffer (0.052 mM, pH 7.0), 0.3 ml of supernatant after centrifugation (1500 xg, 10 min followed by 10,000 × g, 15 min) of 10% homogenate was added to the reaction mixture. Enzyme reaction was initiated by adding 0.2 ml of NADH (780 μM) and stopped after 1 min by adding 1 ml of glacial acetic acid. Amount of chromogen formed was measured by recording color intensity at 560 nm. Results are expressed in units/mg protein.

### Reduced glutathione assay (GSH)

Reduced glutathione was estimated by the method of Jollow et al. [20]. 1.0 ml sample of 10% homogenate was precipitated with 1.0 ml of (4%) sulfosalicylic acid. The samples were kept at 4ºC for 1 hr and then centrifuged at 1200 × g for 20 min at 4ºC. The total volume of 3.0 ml assay mixture composed of 0.1 ml filtered aliquot, 2.7 ml phosphate buffer (0.1 M, pH 7.4) and 0.2 ml DTNB (5,5-dithiobis-2-nitrobenzoic acid), (100 mM). The yellow color of the mixture was developed, read immediately at 412 nm on a Smart SpecTM plus Spectrophotometer and expressed as μM GSH/g tissue.

### Glutathione-*S*-transferase assay (GST)

Glutathione-*S*-transferase activity was assayed by the method of Habig et al. [21]. The reaction mixture consisted of 1.475 ml phosphate buffer (0.1 M, pH 6.5), 0.025 ml (CDNB) (1 mM), 0.2 ml reduced glutathione (1 mM), and 0.3 ml of 10% homogenate in a total volume of 2.0 ml. The changes in the absorbance were recorded at 340 nm and enzymes activity was calculated as nM CDNB conjugate formed/min/mg protein using a molar extinction coefficient of 9.6 × 10^3^/M cm.

### Glutathione reductase assay (GSR)

Glutathione reductase activity was determined by method of Carlberg and Mannervik [22]. The reaction solution composed of 1.65 ml phosphate buffer: (0.1 M, pH 7.6), 0.1 ml EDTA (0.5 mM), 0.1 ml NADPH (0.1 mM) 0.05 ml oxidized glutathione (1 mM), and 0.1 ml 10% homogenate in a total volume of 2 ml. Enzyme activity was quantitated at 25 ºC by measuring disappearance of NADPH at 340 nm and was calculated as nM NADPH oxidized/min/mg protein using molar extinction coefficient of 6.22 ×10^3^/M cm.

### Glutathione peroxidase assay (GSH-Px)

Glutathione peroxidase activity was assayed by the method of Mohandas et al. [23]. The reaction mixture consisted of 1.49 ml phosphate buffer (0.1 M, pH 7.4), 0.1 ml sodium azide (1 mM), 0.05 ml glutathione reductase (1 IU/ml), 0.05 ml GSH (1 mM) 0.1 ml EDTA (1 mM), 0.1 ml NADPH (0.2 mM), 0.01 ml H_2_O_2_ (0.25 mM) and 0.1 ml 10% homogenate in a total volume of 2 ml. The disappearance of NADPH at 340 nm was recorded at 25ºC. Enzyme activity was calculated as nM NADPH oxidized/min/mg protein using molar extinction coefficient of 6.22 × 10^3^/M cm.

### Estimation of lipid peroxidation assay (TBARS)

The assay for lipid peroxidation was carried out following the method of Iqbal et al. [24]. The reaction mixture in a total volume of 1.0 ml contained 0.58 ml phosphate buffer (0.1 M, pH 7.4), 0.2 ml homogenate sample, 0.2 ml ascorbic acid (100 mM), and 0.02 ml ferric chloride (100 mM). The reaction mixture was incubated at 37ºC for 1 h in a shaking water bath. The reaction was stopped by addition of 1.0 ml 10% trichloroacetic acid. After addition of 1.0 ml 0.67% thiobarbituric acid, all the tubes were boiled in a water-bath for 20 min and then shifted to crushed ice-bath before centrifuging at 2500 × g for 10 min. The amount of TBARS formed in each of the samples was assessed by measuring optical density of the supernatant at 535 nm using spectrophotometer against a reagent blank. The results were expressed as nM TBARS/min/mg tissue at 37ºC using molar extinction coefficient of 1.56 × 10^5^/M cm.

### *In Vivo* AChE assessment

AChE activity was determined using the colorimetric assay of Ellman et al. [17], as previously described. Briefly, in the 96 well plates, 25 μl of 15 mM ATCI, 75 μl of 3 DTNB and 75 μl of 50 mM Tris–HCl, pH 8.0, containing 0.1% BSA, were added and absorbance was read at 405 nm after five min incubation at room temperature. Any increase in absorbance due to the pontaneous hydrolysis of the substrate was corrected by subtracting the rate of the reaction before adding the enzyme. Then, 25 μl of sample (SS and DS fraction of brain homogenates) was added, and the absorbance was read again after 5 min ofincubation at room temperature. The AChE activity is expressed as mol/min/g of tissue protein. All determinations were carried out twice and in triplicate.

### Statistical analysis

Computer software SPSS 13.0 was used to determine the level of probability at LSD 0.05%.

## Results

### Effect of LPME on AChE activity (*in vitro* study)

**Figure 1:**
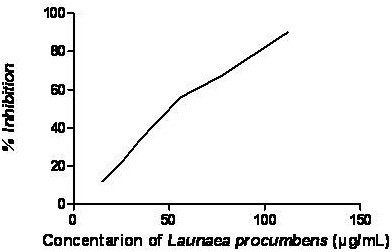
**Effect of LPME on *in vitro *AChE activity**.

To access the *in vitro* efficiency of LPME in inhibiting AChE, different concentrations of LPME (5–150 μg/ml) were used: the results are presented in Figure 1. LPME exhibited moderate AChE inhibitory activity (48 μg/ml).

### Effect of LPME on the step-through test learning ability

Behavioral changes in rats were measured with the administration of LPME (Table 1). Significant changes were not observed during IL measurement in the control (28±3), 100 mg/kg b.w. LPME (24±3) and 200 mg/kg b.w. LPME (27±3) respectively. However, 200 mg/kg b.w. LPME markedly increased (*p* = 0.05) STL compared with the untreated control.

**Table 1:** Effect of LPME on IL and STL

**Treatment**	**IL**	**STL**
Control	28±3	101±32
100 mg/kg b.w. LPME	24±3	108±50
200 mg/kg b.w. LPME	28±7	218±50*

The effect of orally administration of 100 mg/ kg b.w. and 100 mg/kg b.w. LPME extract on initial latency (IL) and step-through latency (STL) in the double trial step-through test. Asterisk (*) indicates significant difference from the control littermates (*n* = 10/group).

### Effect of LPME on body and tissue weight

Body weight of all rats were checked before experiment and after experiment, found non significant changes. Similarly no considerable differences (*p* > 0.05) were found in tissue weight (whole brain) between non treated rats and LPME treated rats (Table 2).

**Table 2:** Showing body weight before and after treatment and wet brain weight

**Treatment**	**Control**	**100 mg/kg b.w. LPME**	**200 mg/kg b.w. LPME**
Body weight before treatment (g)	186.2±3.4	183.6±4.2	188.2±7.9
Body weight after treatment (g)	200.0±5.4	189.5±3.8	195.8±7.2
Wet brain weight (mg)	362.2±3.5	361.5±5.1	357.1±3.4

Mean±S.E. values (*n* = 10/group). Control group received orally of saline.

### Effect of LPME on brain AChE activity

Table 3 shows the activity of AChE in DS and SS homogenate of rats. Administration of 100 and 200 mg/kg b.w., considerably improved (*p* < 0.05) AChE in DS and SS homogenate as comparatively to non treated rats. Remarkable inhibition (*p* < 0.05) was found in the brain tissue of 200 mg/kg b.w., LPME (74% in DS and 71% in SS) comparative to 100 mg/kg b.w., LPME (42% in DS and 31% in SS) treated rats.

**Table 3:** **Effect of LPME on *ex vivo *AChE activity (mol/min/g of tissue protein) in rat brain**

**Treatment**	**Salt soluble (SS)-AChE**	**Detergent soluble (DS)-AChE**
Control	0.175 ± 0.0057	0.889 ± 0.074
100 mg/kg b.w. LPME	0.101 ± 0.002*	0.525 ± 0.031**
200 mg/kg b.w. LPME	0.74 ± 0.010**	0.300 ± 0.051**

a The AChE activity for each group denotes mean±S.E.M values.

* *p* < 0.05 significant difference from control.

** *p* < 0.01 significant difference from control.

### Effect of LPME on brain oxidative status

Alteration in the activity of SOD, CAT, GSH, GSHpx, GST, GSR and TBARS are shown with administration of LPME in rats in a dose-dependent way (Table 4). In particular, 200 mg/kg b.w., of LPME administration markedly *(p<0.05)* reduced TBARS while considerably improved (*p* < 0.05) activities of GSH, GST, GSR and GSHpx were significantly (*p* < 0.05) in the cerebral tissue of rats to that of non treated control rats.

**Table 4:** Effect of LPME on biochemical parameters of rat brain antioxidant status

**Treatment**	**CAT (U/min)**	**SOD (U/mg protein)**	**GSHpx nmol/mg protein)**	**GSR (nmol/min/mg protein)**	**GST (nmol/min/mg protein)**	**GSH (μmol/g tissue)**	**TBARS (nmol/min/mg protein)**
Control	11.0±0.25	7.18±2.8	43.3±3.58	174.5±20.7	115.8±26.11	64.5±10.7	184.5±8.7
100 mg/kg b.w. LPME	15.5±2.8*	13.5±1.4**	55.3±1.85*	203.3±10.3**	139.8±13.3 *	88.0±8.3*	156.3±6.3*
200 mg/kg b.w. LPME	14.0±1.12*	18.3±1.7 **	65.3±5.14**	218.0±10.5 **	150.8±11.5**	91.8±10.0**	144.0±5.5**

a The rats brain biochemical parameters are expressed as mean±S.E.M values.

* *p* < 0.05 significant difference from control littermates (*n* = 10/group).

** *p* < 0.01 significant difference from control littermates (*n* = 10/group).

## Discussion

Medicinal plants and their bioactive fractions have significant roles in various disorders. These fractions have been shown to improve the learning behaviors and memories of experimental animals [16]. For determination of disorders of the central nervous system in experimental animals, a passive avoidance task (fear-aggravated test) is used for evaluation of learning and memory. In the present study, on the 5th day, rats were placed in a dark chamber to evaluate their vision and neuronal activity. The results of the present study revealed that the values of STL differed significantly, whereas no change was found in IL values. Similar findings were reported by Ellman *et al.*[15] in their study on *Vaccinium ashei* supplementation in mice. Our results suggested that administration of *Launaea procumbens* significantly improved memory and learning in experimental animals when using passive avoidance tests. Similar results have been reported in other experimental animals [16]. The data of the present study revealed that LPME administration in rats significantly decreased brain AChE activity. This phenomenon might be due to decreases in the transcription and translation of genes as well as enhanced cholinergic activity, which improves cognitive function [25]. AChE activity was reduced, with significant effects in rats treated with 200 mg/kg b.w. LPME compared with the untreated group. Oxidative stress and the antioxidant system have important roles in the pathophysiology of cerebral changes and brain disorders. Activities of superoxide dismutase (SOD) and catalase (CAT) are susceptible to oxidative changes. SOD and CAT have important roles in the defense against oxidative stress. They reduce hydrogen peroxide and prevent the generation of hydroxyl radicals, thereby shielding cellular constituents from oxidative damage. The present study revealed that administration of 100 mg/kg b.w. LPME and 200 mg/kg b.w. LPME increased the activity of SOD and CAT, as reported during supplementation of *Launaea procumbens* in rats [2]. GSH provides the first line of defense for the body by scavenging reactive oxygen species (ROS). The decreased concentration of GSH in the liver might be due to NADPH reduction or GSH utilization in the exclusion of peroxides [26]. GSH-dependent enzymes offer a second line of defense because they detoxify noxious byproducts generated by ROS and also help to avert the dissemination of free radicals [27]. GSH-Px detoxifies peroxides by reacting with GSH and converting it to GSSG, which is reduced to GSH by GSR [28]. GSHpx, glutathione reductase (GSR) and glutathione-S-transferase are basic antioxidant enzymes. Therefore, the profile of these enzymes and their alteration are a strong link with neurodegenerative diseases. Supplementation with *Launaea procumbens* for 7 days improved the activity of these basic antioxidant enzymes, showing protection against free radicals. From these results it was inferred that LPME administration in healthy rats significantly attenuated oxidative damage in the brain, increased the activity of antioxidant enzymes, increased GSH contents, increased the activity of AChE, and decreased the level of TBARS. These effects were related to enhancement in the passive avoidance tests. Despite these findings, further research on the mechanisms involved in this process is in progress.

## Conclusion

From the present result it is inferred that LPME is beneficial for improving the learning ability and antioxidant potential.

## Abbreviations

AChE: Acetylcholinesterase; ATCI: Acetyl thiocholine iodide; b.w.: Body weight; BSA: Bovine serum albumin; DS: Detergent soluble; DMSO: Dimethyl sulfoxide; DTNB: 5,5 dithiobis-2-nitrobenzoate ion; EDTA: Ethylenediaminetetraacetic acid; GSHpx: Glutathione peroxidase; GSH: Glutathione; GST: Glutathione *S*-transferase; IC50: Median inhibition concentration; IL: Initial latency; SS: Salt soluble fraction; STL: Step-through latency; TBA: Thio barbituric acid; TBARS: Thio barbituric acid reactive substances; Tris–HCl: Tris famino methane hydrochloride.

## Competing interests

The authors declare that they have no competing interests.

## Author’s contributions

RAK made a significant contribution to acquisition of data, analysis, drafting of the manuscript and in revising the manuscript for intellectual content. The author read and approved the final manuscript.

## Pre-publication history

The pre-publication history for this paper can be accessed here:

http://www.biomedcentral.com/1472-6882/12/219/prepub

## References

[B1] Parikh J Chanda S Screening of aqueous and alcoholic extracts of some Indian medicinal plants for antibacterial activity Indian J Pharm Sci 2006 68 835 838 10.4103/0250-474X.31032

[B2] Khan RA Khan MR Sahreen S Evaluation of *Launaea procumbens* use in renal disorders: a rat model J Ethnopharmacol 2010 128 452 461 10.1016/j.jep.2010.01.026 20096342

[B3] Ahmad M Khan MA Manzoor S Zafar M Sultana S Check list of medicinal flora of Tehsil Isakhel, District Mianwali Pakistan Ethno Leaflets 2006 10 41 48

[B4] Qureshi R Bhatti GR Ethnobotany of plants used by the Thari people of Nara Desert, Pakistan Fitoterapia 2008 79 468 473 10.1016/j.fitote.2008.03.010 18538950

[B5] Khan RA Khan MR Sahreen S Attenuation of CCl4-induced hepatic oxidative stress in rat by *Launaea procumbens* Exp Toxicol Pathol 2011 http://dx.doi.org/10.1016/j.etp.2011.11.001. 10.1016/j.etp.2011.11.001 22134123

[B6] Shaukat SS Siddiqui IA Nasim AI Nematocidal, allelophatic and antifugal potential of *Launaea procumbens* Pakistan J Plant Pathol 2003 2 181 193

[B7] Middleton C Kandaswami TC Theoharides TC The effects of plant flavonoids on mammalian cells: implications for inflammation, heart disease, and cancer Pharmacol Rev 2000 52 673 751 11121513

[B8] Balasundram N Sundram K Samman S Phenolic com-pounds in plants and agri-industrial by-products: Antioxidant activity, occurrence, and potential uses Food Chem 2006 99 191 203 10.1016/j.foodchem.2005.07.042

[B9] Zhou T Luo D Li X Luo Y Hypoglycemic and hypolipidemic effects of flavonoids from lotus (*Nelumbo nuficera* Gaertn) leaf in diabetic mice J Med Plants Res 2009 3 290 293

[B10] Joseph JA Denisova NA Arendash G Gordon M Diamond D Shukitt-Hale B Blueberry supplementation enhances signaling and prevents behavioral deficits in an Alzheimer disease model Nutr Neurosci 2003 6 153 162 10.1080/1028415031000111282 12793519

[B11] Shukitt-Hale B Lau FC Joseph JA Berry fruit supplementation and the aging brain J Agric Food Chem 2008 56 636 641 10.1021/jf072505f 18211020

[B12] Sarter M Bruno JP Cognitive functions of cortical acetylcholine: toward a unifying hypothesis Brain Res Rev 1997 23 28 46 10.1016/S0165-0173(96)00009-4 9063585

[B13] Zimmerman G Soreq H Termination and beyond: acetylcholinesterase as a modulator of synaptic transmission Cell Tissue Res 2006 326 655 669 10.1007/s00441-006-0239-8 16802134

[B14] Lane RM Potkin SG Enz A Targeting acetylcholinesterase and butyrylcholinesterase in dementia Intl J Neuropsycopharmacol 2006 9 101 124 10.1017/S1461145705005833 16083515

[B15] Ellman GL Courtney KD Andres V Featherstone RM A new and rapid colorimetric determination of acetylcholinesterase activity Biochem Pharmacol 1996 7 88 95 13726518 10.1016/0006-2952(61)90145-9

[B16] Kameyama T Nabeshima T Kozawa T Step-down-type passive avoidanceand escape-learning method. Suitability for experimental amnesia models J Pharmacol Methods 1986 16 39 52 10.1016/0160-5402(86)90027-6 3747545

[B17] Khan RA Khan MR Sahreen S Brain antioxidant markers, cognitive performance and acetyl cholinesterase activity of rat: Efficiency of *Sonchus asper* Behavioral and Brain Functions 2012 8 21 10.1186/1744-9081-8-21 22591917 PMC3527136

[B18] Chance B Maehly AC Assay of catalase and peroxidases Methods Enzymol 1955 11 764 775

[B19] Kakkar P Das B Viswanathan PN A modified spectrophotometric assay of superoxide dismutase Indian J Biochem Biophys 1984 21 130 132 6490072

[B20] Jollow DJ Mitchell JR Zampaglione N Gillete JR Bromobenzene induced liver necrosis. Protective role of glutathione and evidence for 3, 4-bromobenzene oxide as a hepatotoxic metabolite Pharmacol 1974 11 151 169 10.1159/000136485 4831804

[B21] Habig WH Pabst MJ Jakoby WB Glutathione-S-transferases: the first enzymatic step in mercapturic acid formation J Biol Chem 1974 249 7130 7139 4436300

[B22] Carlberg I Mannervik EB Glutathione level in rat brain J Biol Chem 1975 250 4475 4480 237922

[B23] Mohandas J Marshal JJ Duggin GG Horvath JS Tiller DJ Differential distribution of glutathione and glutathione-related enzymes in rabbit kidney. Possible implications in analgesic nephropathy Biochem Pharmacol 1984 33 1801 1807 10.1016/0006-2952(84)90353-8 6145422

[B24] Iqbal M Sharma MD Zadeh HR Hasan N Abdulla M Athar M Glutathione metabolizing enzymes and oxidative stress in ferric nitrilotriacetate (Fe-NTA) mediated hepatic injury Redox Report 1996 2 385 391 10.1080/13510002.1996.11747079 27406673

[B25] Shahidi S Komaki A Mahmoodi M Atrvash N Ghodrati M Ascorbic acid supplementation could affect passive avoidance learning and memory in rat Brain Res Bull 2008 76 109 113 10.1016/j.brainresbull.2008.01.003 18395619

[B26] Yadav P Sarkar S Bhatnagar D Action of *Capparis deciduas* against alloxan-induced oxidative stress and diabetes in rat tissues Pharmacol Res 1997 36 221 228 10.1006/phrs.1997.0222 9367667

[B27] Gumieniczek A Effects of repaglinide on oxidative stress in tissues of diabetic rabbits Diab Res Clin Pract 2005 68 89 95 10.1016/j.diabres.2004.09.018 15860235

[B28] Maritim AC Sanders RA Watkins JB Effects of α-lipoic acid on biomarkers of oxidative stress in streptozotocin-induced diabetic rats J Nutr Biochem 2003 14 288 294 10.1016/S0955-2863(03)00036-6 12832033

